# Estimating Metastatic Risk of Pancreatic Ductal Adenocarcinoma at Single-Cell Resolution

**DOI:** 10.3390/ijms232315020

**Published:** 2022-11-30

**Authors:** Sina Chen, Shunheng Zhou, Yu-e Huang, Mengqin Yuan, Wanyue Lei, Jiahao Chen, Kongxuan Lin, Wei Jiang

**Affiliations:** Department of Biomedical Engineering, Nanjing University of Aeronautics and Astronautics, Nanjing 211106,China

**Keywords:** single-cell RNA sequencing, spatial transcriptomics, pancreatic ductal adenocarcinoma, metastasis, drug prediction

## Abstract

Pancreatic ductal adenocarcinoma (PDAC) is characterized by intra-tumoral heterogeneity, and patients are always diagnosed after metastasis. Thus, finding out how to effectively estimate metastatic risk underlying PDAC is necessary. In this study, we proposed scMetR to evaluate the metastatic risk of tumor cells based on single-cell RNA sequencing (scRNA-seq) data. First, we identified diverse cell types, including tumor cells and other cell types. Next, we grouped tumor cells into three sub-populations according to scMetR score, including metastasis-featuring tumor cells (MFTC), transitional metastatic tumor cells (TransMTC), and conventional tumor cells (ConvTC). We identified metastatic signature genes (MSGs) through comparing MFTC and ConvTC. Functional enrichment analysis showed that up-regulated MSGs were enriched in multiple metastasis-associated pathways. We also found that patients with high expression of up-regulated MSGs had worse prognosis. Spatial mapping of MFTC showed that they are preferentially located in the cancer and duct epithelium region, which was enriched with the ductal cells’ associated inflammation. Further, we inferred cell–cell interactions, and observed that interactions of the ADGRE5 signaling pathway, which is associated with metastasis, were increased in MFTC compared to other tumor sub-populations. Finally, we predicted 12 candidate drugs that had the potential to reverse expression of MSGs. Taken together, we have proposed scMetR to estimate metastatic risk in PDAC patients at single-cell resolution which might facilitate the dissection of tumor heterogeneity.

## 1. Introduction

Pancreatic cancer (PC) is one of the most devastating cancers, whose five-year survival rate is less than 5%. Approximately 90% of PC patients are pancreatic ductal adenocarcinoma (PDAC) [1,2]. PDAC has a high capacity for metastatic dissemination to other organs, which will lead to poor prognosis [3]. Surgical resection remains the only potentially curative therapy. However, tumor cells have already distally metastasized when most patients are diagnosed, in which case surgical therapy is not recommended [4].

Tumor cell distal metastasis is a multi-step process including local invasion, intravasation, survival in the blood circulation, extravasation, adaptation to survival in the new microenvironment, and finally colonization and outgrowth in distant organs [5,6]. Epithelial-mesenchymal transition (EMT) plays an important role in metastasis. During EMT, cells will increase their migratory capacity and lose their epithelial cell–cell adherens junction. In distant organs, mesenchymal-epithelial transition (MET), which is the reversion of EMT, is apparent. MET will make tumor cells return to an epithelial morphology, which is beneficial to PDAC cells invading the surrounding tissues [1,4,7,8]. Therefore, metastasis-featuring cells may exist in primary tissue and distant organs. A prior study reported that the differentiation status of tumor cells is also related with metastasis. An immature tumor is more aggressive than its more differentiated counterpart. Poorly differentiated tumors tend to spread faster than well-differentiated tumors [9,10]. PDAC displays intra-tumoral heterogeneity in distinguishable features, which makes it rather challenging to evaluate its metastatic risk.

Single-cell RNA sequencing (scRNA-seq) is a powerful technique to reveal intra-tumoral heterogeneity and could be used for exploring the unbiased and systematic characterization of the distinct cell populations that present in tumor tissue [11,12,13]. Some studies evaluated the metastatic risk of a tumor based on clinical, histological and genomic features [14,15]. However, those studies could not be applied to single-cell transcriptomics.

**Figure 1:**
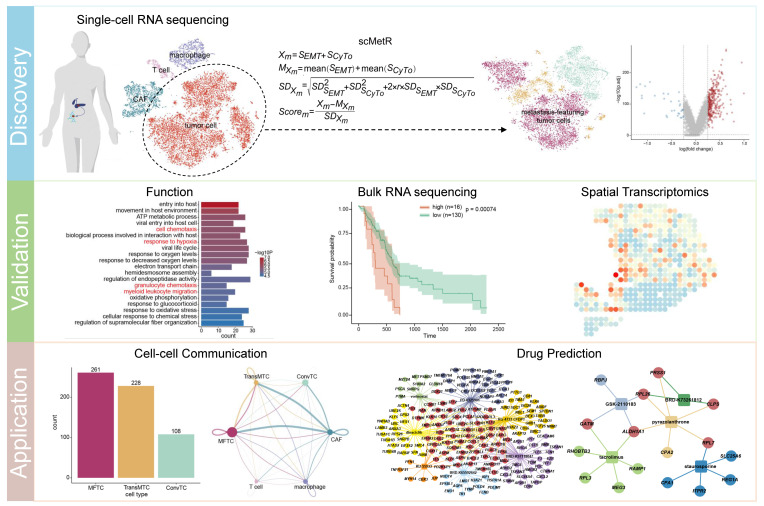
Analysis workflow for pancreatic ductal adenocarcinoma (PDAC) data.

A previous study profiled primary and metastatic PDAC using scRNA-seq [16]. By conducting an analysis of this dataset, we identified major cell types in PDAC and dissected the PDAC tumor heterogeneity. We established scMetR to estimate the metastatic risk of tumor cells, and found heterogeneous sub-populations in tumor cells with different metastatic risks. Further, we identified metastatic signature genes (MSGs) and validated the metastatic risk of tumor sub-populations from multiple perspectives, including functional enrichment, prognosis of patients, spatial expression patterns of metastasis-associated genes, spatial location of tumor sub-populations, and cell–cell communication. The above discoveries were reproducible in an independent scRNA-seq dataset. Finally, through drug prediction based on MSGs, we found some candidate inhibitors of metastasis (Figure 1).

## 2. Results

### 2.1. Major Cell Types in PDAC Primary and Metastatic Patients

**Figure 2:**
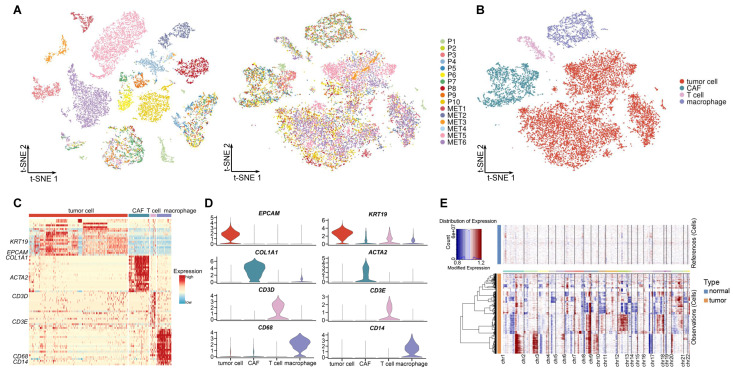
Cell composition of pancreatic ductal adenocarcinoma (PDAC). (**A**) t-distributed stochastic neighbor embedding (t-SNE) plot for distinct patients. Cells are colored according to patients before (left) and after (right) integration. (**B**) t-SNE plot of 4 major cell types identified in primary and metastatic PDAC. (**C**) Heatmap of specific markers in each cell type. (**D**) Violin plots of expression for well-known markers. The *y*-axis shows the expression level. (**E**) Heatmap of inferred copy number variation (CNV) across normal epithelial cells and tumor cells.

To explore the cellular diversity in primary and metastatic samples, we accessed a published dataset, which profiled 10 primary patients and 6 metastatic patients using scRNA-seq [16]. After initial quality control, we acquired single-cell transcriptomes in a total of 8201 cells from primary samples and 7332 cells from metastatic samples. We applied a principal component analysis (PCA) on highly variable genes (HVGs) across all cells. Then, cells were visualized by t-distributed stochastic neighbor embedding (t-SNE). We found that the cells were grouped primarily by samples, which suggested that the batch effects between samples existed in the cells (Figure 2A) and would confound key biological variations of PDAC. Therefore, we integrated all samples to correct the batch effects. Then, we checked the sample distribution of the integrated data and found an unbiased distribution of most cells (Figure 2A and Figure S1A). Unsupervised clustering using Seurat (V3.2.2) revealed seven clusters (Figure S1B), which were annotated as tumor cell, cancer associated fibroblast (CAF), T cell, and macrophage (Figure 2B). To define the identity of each cell cluster, we generated cluster-specific marker genes by performing a differential gene expression analysis. Some well-known marker genes were used to identify cell types, such as *EPCAM* and *KRT19* for tumor cell, *COL1A1* and *ACTA2* for CAF, *CD3D* and *CD3E* for T cell, and *CD68* and *CD14* for macrophage [12,16,17] (Figure 2C,D and Figure S1C,D). The marker genes of different cell types contained those known maker genes which had high expression levels in the corresponding cell types. This suggested that the annotation of cells was right. To further verify the assignment of tumor cells, we performed an inferred gene copy number analysis using inferCNV [18]. When using the expression profiles of normal epithelial cells from the Genome Sequence Archive (GSA) as reference, the copy number profiles inferred from the tumor cells showed substantial copy number variation (CNV) (Figure 2E), which was consistent with the fact that PDAC tumor cells were highly aneuploidy with numerous copy number alterations (CNA) [12,16,19].

### 2.2. Heterogeneity in Tumor Cells

**Figure 3:**
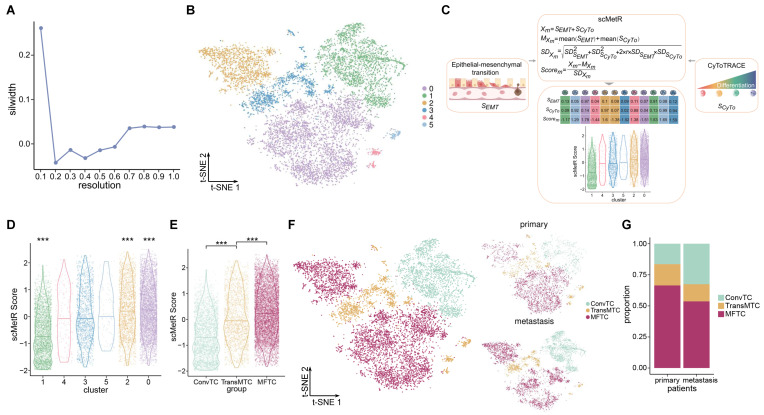
Different metastatic risk of tumor sub-clusters. (**A)** The plot of mean silhouette width (silwidth) at each resolution. (**B)** t-distributed stochastic neighbor embedding (t-SNE) plot of tumor sub-clusters using the resolution of 0.1. (**C**) Schematic description for calculating scMetR score. (**D**) Violin plots of scMetR score for each tumor sub-cluster. (**E**) Violin plots of scMetR score for each tumor sub-population. (**F**) t-SNE plot of tumor sub-populations in the primary and metastatic samples, which are identified based on scMetR. Top right: the primary samples. Bottom right: the metastatic samples. (**G**) Bar plot of proportion in primary and metastatic patients for tumor sub-populations. *** *p* < 0.001 using the Wilcoxon test.

It is appreciated that PDAC could be composed of multiple sub-populations of tumor cells which differ among themselves in many properties, such as metastatic risk. To explore the heterogeneity of tumor cells, we re-clustered the tumor cells into sub-clusters by a resolution which was selected based on mean silhouette width (silwidth) [20]. Silwidth represented the similarity of a cluster and the difference of distinct clusters. A larger value of silwidth meant that the cells of same cluster had a higher similarity. We calculated the mean silwidth at varying resolutions from 0.1 to 1 by the steps of 0.1 and observed that the mean value of silwidth was the largest at 0.1 (Figure 3A). Finally, the tumor cells were divided into six sub-clusters based on the resolution of 0.1 (Figure 3B). To estimate the metastatic risk of each sub-cluster, we calculated $S_{EMT}$ and $S_{CyTo}$, where $S_{EMT}$ is a score to evaluate the expression of genes associated with EMT [21,22] and $S_{CyTo}$ is a score to assess the differential potential for tumor cells [23,24]. A previous study found that well-differentiated tumor cells were more similar to normal cells and tended to grow and spread more slowly than poorly differentiated or undifferentiated tumor cells [9,10]. In addition, EMT was correlated with metastasis as well [8]. In order to accurately assess the metastatic risk of tumor cell sub-clusters, we proposed a metastatic risk score, named scMetR, by integrating $S_{EMT}$ and $S_{CyTo}$ (Figure 3C). The results displayed that the scMetR scores of sub-clusters 0, 1, and 2 were significantly different (Wilcoxon test: *p* < 0.01; Figure 3D and Figure S2A). Thus, three sub-populations with distinct metastatic risk were identified. We defined sub-clusters 0 and 2 as metastasis-featuring tumor cells (MFTC); sub-clusters 3, 4, and 5 as transitional metastatic tumor cells (TransMTC); and sub-cluster 1 as conventional tumor cells (ConvTC). We compared the scMetR score among tumor sub-populations and observed that the scMetR score was significantly increased in MFTC compared to TransMTC, and in TransMTC compared to ConvTC. Figure 3E). Then, we visualized the number and proportion of tumor sub-populations in primary and metastatic samples. Although MFTC was more numerous in metastatic samples, their proportion was higher in primary samples (Figure 3F,G and Figure S2B).

### 2.3. Signatures Associated with Metastasis

**Figure 4:**
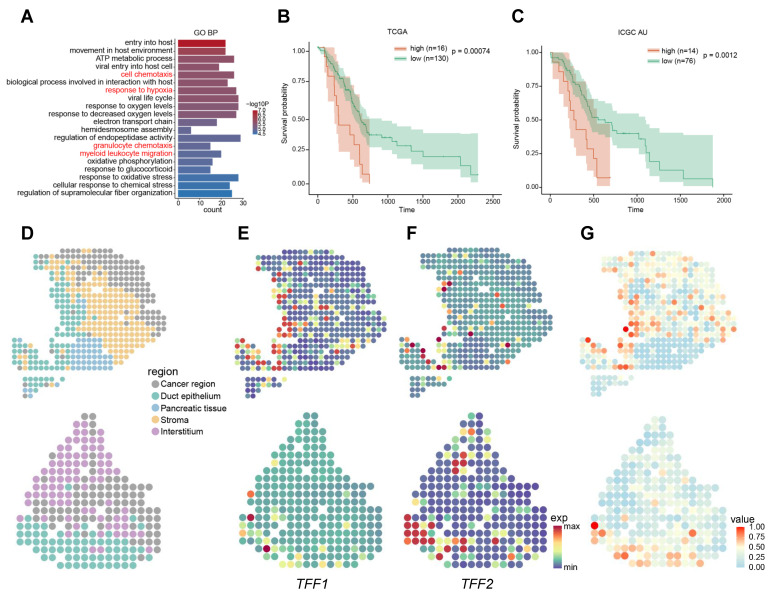
Analysis of up-regulated metastatic signature genes (MSGs). (**A**) Bar graph of the top 20 significant Gene Ontology (GO) terms, ordered by decreasing −$\log_{10}$*p* value. The *x*-axis shows the number of genes enriched for the corresponding GO term. The terms which were colored red were associated with metastasis. (**B**,**C**) Kaplan–Meier survival curves for patients in The Cancer Genome Atlas (TCGA) and AU cohort of International Cancer Genome Consortium (ICGC) datasets. (**D**) The unsupervised clustering analysis of spatial transcriptomics (ST) in pancreatic ductal adenocarcinoma (PDAC). Colors indicate the distinct regions. (**E**,**F**) Spatially resolved heatmaps of expression patterns for *TFF1* and *TFF2*. (**G**) Spatially resolved heatmaps of proportion of metastasis-featuring tumor cells (MFTC). The proportion is indicated by the colors.

To reveal the effect of metastasis in cancer, we conducted further analyses. We found the differentially expressed genes (DEGs) between MFTC and ConvTC and defined them as MSGs, which included 431 genes (Table S1). To understand the potential related functions, we applied functional enrichment analysis on up-regulated MSGs and identified several functions associated with metastasis, including cell chemotaxis [25], response to hypoxia [26,27], granulocyte chemotaxis [28,29], and myeloid leukocyte migration [29,30] (Figure 4A). These meant that up-regulated MSGs might promote metastasis. It was reported that the clinical outcome of metastatic PDAC patients was poorer than that of primary PDAC patients [5,8], which suggested that the survival of patients with highly metastatic risk was poorer than that of patients with low metastatic risk. Thus, we used the survival data of patients from The Cancer Genome Atlas (TCGA) and the International Cancer Genome Consortium (ICGC) databases to analyze patient prognosis to validate whether the MFTC had a high risk of metastasis. The patients that had high expression of up-regulated MSGs might have high metastatic risk. Therefore, a single sample gene set enrichment analysis (ssGSEA) [21] was performed to evaluate the expression level of up-regulated MSGs, and patients were categorized into high or low groups based on their ssGSEA scores. The results of the survival analysis indicated that the patients with a high expression of up-regulated MSGs had shorter survival (Figure 4B,C and Figure S2C). Further, we analyzed a published dataset which profiled two primary patients by spatial transcriptomics (ST) to explore the spatial characteristic of MFTC [31]. ST data were classified into region by PCA on the HVGs. After unsupervised clustering the spots, we found that the identified clusters were consistent with well-defined histological annotations (Figure 4D) [31]. We observed that some up-regulated MSGs were verified to promote PDAC cell migration in previous studies, such as *TFF1*, *TFF2*, and *HMGA1* [32,33,34]. They were highly expressed in duct epithelium region and cancer region (Figure 4E,F and Supplementary Note, Figure S4). MFTC was found to be preferentially located in the duct epithelium and cancer region by mapping cell types in PDAC slides (Figure 4G). Moncada et al. reported that duct cells associated with inflammation were enriched in duct epithelium region [31]. These results show that metastasis-associated genes were expressed highly in the duct epithelium region and MFTC was mainly distributed in the duct epithelium region which were consistent with prior reports on inflammation-promoting PDAC metastasis [35,36,37].

### 2.4. Cell–Cell Communications in PDAC

**Figure 5:**
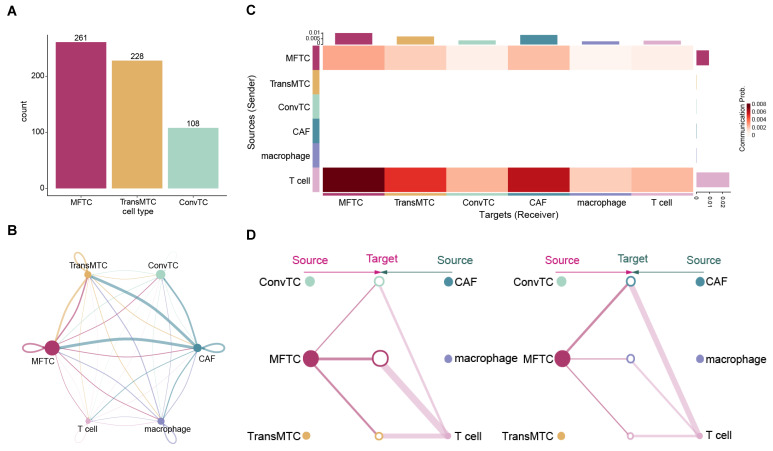
Cell–cell communication of major cell types and tumor sub-populations. (**A**) The total number of interactions from tumor sub-populations. (**B**) The number of interactions among distinct cell types. The thickness of the lines connecting cells indicates the number of interactions. The size of the circle indicates the number of cells. (**C**) Heatmap of the interaction between any two cell populations in the ADGRE5 signaling pathway. Bar plots on the right and top represent the total outgoing and incoming signaling strength, respectively. (**D**) The inferred ADGRE5 signaling network. The left and right plots display the autocrine and paracrine signaling to tumor sub-populations and other cell types, respectively. Solid and open circles represent the source and the target, respectively. The size of the circle indicates the number of cells. The thickness of the lines connecting cells indicates the interaction strength.

In addition to the intrinsic cell information, cell–cell communication might also contribute to promoting metastasis. We employed CellChat (V1.5.0) [38] to study the signaling interactions among all major cell types and sub-populations of tumor cells. We compared the total number and the strength of interactions of tumor sub-populations that were inferred by CellChat in PDAC. We found that the total number and the strength of interactions were the highest in MFTC and second highest in TransMTC (Figure 5A,B and Figure S3A,B). To identify the signaling pathways contributing to metastasis, 206 significant ligand-receptor interactions within 41 signaling pathways were predicted, including ADGRE5, COLLAGEN, SPP1, and ICAM (Figure S3C). A previous study demonstrated that activation of ADGRE5 signaling was associated with lymph node involvement, metastasis, and vascular invasion [39]. Indeed, the inferred cell–cell communication network of the ADGRE5 signaling pathway showed that the interaction strength was highest in MFTC among the tumor sub-populations and second highest in TransMTC (Figure 5C,D and Figure S3D). These results show that total number and strength of interactions and the interaction of the ADGRE5 signaling pathway were enhanced in MFTC compared to the other tumor sub-populations, suggesting that MFTC had the highest risk of metastasis among tumor sub-populations, which was consistent with the scMetR score of the tumor sub-populations.

### 2.5. Potential Inhibitors Targeting the Signatures Associated with Metastasis

**Figure 6:**
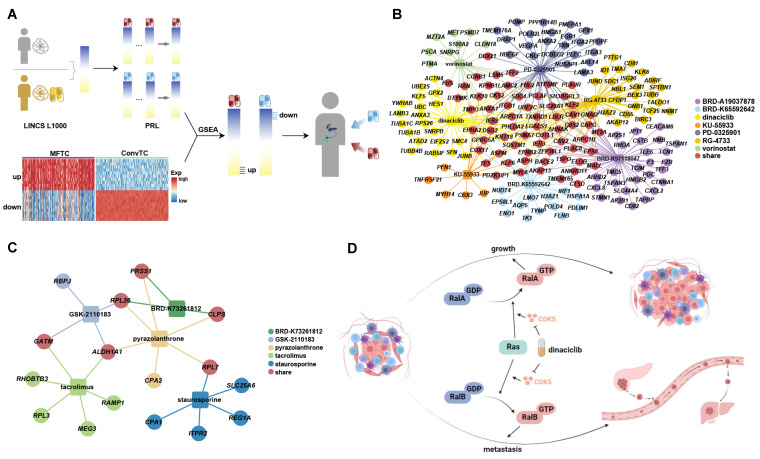
Drug prediction based on metastatic signature genes (MSGs). (**A**) Workflow of drug prediction. (**B**,**C**) Network of genes regulated by each drug. Drugs regulating the top 10% of genes from up- (**B**) or down-regulated (**C**) MSGs are displayed. Rectangles indicate drugs, and circles indicate genes. Each line and node color indicates one drug. If a gene could be regulated by more than one drug, the node is colored red. (**D**) Depiction of the regulation of cyclin-dependent kinase 5 (CDK5) by dinaciclib in tumor growth and metastasis. Created with BioRender.com on 2 September 2022.

Surgical resection is the only possibly curative treatment for PDAC, but distant recurrence after the surgical resection of PDAC is common [5]. Therefore, it is necessary to find inhibitors of metastasis. To find potentially therapeutic drugs, data from The Library of Integrated Network-based Cellular Signatures (LINCS) representing gene expression changes before and after drug treatment were analyzed. Using gene set enrichment analysis (GSEA) [40], we tested whether MSGs were present among the genes which were up- or down-regulated by a drug. If up-regulated MSGs were present among the genes which were down-regulated by a drug, it suggested that the drug might reverse the expression of the up-regulated MSGs and be a potential inhibitor for metastasis (Figure 6A). There were seven inhibitors identified across the up-regulated MSGs, including BRD-A19037878, BRD-K65592642, dinaciclib, KU-55933, PD-0325901, RG-4733, and vorinostat. Across the down-regulated MSGs, five inhibitors were found, including BRD-K73261812, GSK-2110183, pyrazolanthrone, tacrolimus, and staurosporine (Figure 6B,C and Table S2). Among them, dinaciclib had been proved to inhibit the growth and metastasis of pancreatic cancer [41,42,43,44]. Previous studies demonstrated that the Ras-like (Ral) effector pathway, activated by Ras, plays critical and divergent roles in pancreatic cancer [41,42]. RalA and RalB are critical effectors of the Ral pathway [41,42,43]. Activating RalA would promote the growth of tumor cells, and loss of RalB function inhibited tumor metastasis [41,42]. It was reported that cyclin-dependent kinase 5 (CDK5) promoted the Ral effector pathway central to Ras signaling [43]. Thus, dinaciclib which is an inhibitor of CDK5 could block Ras–Ral signaling by inhibiting the activation of CDK5 [44] (Figure 6D). Further, previous study reported that the number of metastatic lesions was reduced in both Panc265 and Panc253, which were patient-derived pancreatic cancer xenograft models, after treating with dinaciclib [45].

## 3. Discussion

PDAC is a heterogeneous disease with a differing metastatic risk in tumor cells. Prior studies reported that metastasis will lead to poor prognosis of patients [5,8]. Thus, it is highly desirable to explore the tumor heterogeneity and the underlying metastatic risk, which are pivotal for PDAC prognostic improvement. In this study, we identified different cell types in PDAC based on scRNA-seq data, including tumor cell, CAF, T cell, and macrophage (Figure 2B). To reveal the tumor heterogeneity, we re-clustered tumor cells using a resolution which was selected based on the mean silwidth. Then, we established scMetR, which integrated the EMT-associated gene score and the score that was used to assess the differential potential for tumor cells to observe the distinct metastatic risk of tumor sub-clusters (Figure 3C). Next, three tumor sub-populations with differing metastatic risk levels, namely MFTC, TransMTC, and ConvTC, were defined based on the scMetR score (Figure 3E,F). The metastatic risk of MFTC was validated by functional enrichment, prognosis of patients, spatial expression patterns of the genes related to metastasis, spatial location of MFTC, and cell–cell communication. The results suggested that MFTC has a high risk of metastasis. To find potential inhibitors of metastatic PDAC, we analyzed LINCS data, which represented gene expression changes following treatment with drugs, and investigated the effect of these drugs for MSGs’ expression based on these data. Finally, 12 potentially therapeutic drugs which might reverse the expression level of MSGs were identified.

In order to evaluate the efficiency of scMetR, we performed the same analyses in an independent scRNA-seq dataset of PDAC (details in Supplementary Note). We found that the up-regulated MSGs were also enriched in the functions associated with metastasis, such as neutrophil degranulation [46], neutrophil mediated immunity [47]; the patients with high expression of the up-regulated MSGs had a worse prognosis; MFTC were preferentially located in duct epithelium region and cancer region; the total number and the strength of cell–cell interactions were the highest in MFTC and the second highest in TransMTC. These observations were consistent with the previous discoveries, which suggested that scMetR was reproducible in the independent dataset.

Our study has several limitations as well. One of the challenges in scRNA-seq data analyses is the occurrence of dropout events, which represent missing data or no gene expression. Dropout events might lead to confusion in downstream analysis [48,49]. There have been several imputation methods to deal with dropout events, such as Markov affinity-based graph imputation of cells (MAGIC) [50]. However, applying imputation methods might not improve the performance of downstream analyses and might introduce false-positives [48,51,52]. Thus, the imputation method was not performed in our analysis. In addition, the single-cell transcriptional profiles which were used for analysis contained 17,086 cells. However, those cells were not sufficient. Increasing the number of cells used for analysis would increase the credibility of subsequent work. Furthermore, the highly metastatic risk of MFTC and the effectiveness of inhibitors still lacked experimental verification, which would provide powerful evidence for this discovery.

Overall, our findings identified major cell types in primary and metastatic PDAC, and we proposed scMetR to reveal tumor heterogeneity regarding metastatic risk. Further analysis suggested that MFTC, which were defined based on scMetR, had a highly metastatic risk. Meanwhile, we predicted potential inhibitors of tumor metastasis. Thus, our analysis might provide a new measurement to estimate metastatic risk, which would be helpful for the diagnosis and treatment of metastasis.

## 4. Materials and Methods

### 4.1. Single-Cell RNA Sequencing Data Processing

We downloaded published PDAC data from the Gene Expression Omnibus (GEO) database (GSE154778 [16]) which included 10 primary samples and 6 metastatic samples. The number of cells obtained from each patient ranged from 585 to 1570 for primary samples and from 272 to 2905 for metastatic samples. The dataset contained 9621 cells of primary samples and 7465 cells of metastatic samples. Genes expressed in fewer than 3 cells were removed. Cells with more than 40% mitochondrial gene counts, more than 7500 detected genes, and more than 100,000 unique molecular identifier (UMI) were filtered [17]. This preprocessing was performed with Seurat (V3.2.2) R package [53].

### 4.2. Identifying Major Cell Types in PDAC

All the cells passing quality control were merged and normalized using the NormalizeData function with default parameters. The top 2000 HVGs were selected using the FindVariableGenes function. Then, these data were scaled using the ScaleData function with default parameters. Harmony was used for batch correction [54]. The FindCluster function was used to find clusters. The function returned several cell clusters which were visualized with t-SNE. The FindMarkers function was used to identify cluster-specific marker genes and marker genes of the distinct cell types. Adjusted *p* < 0.05 and |logFC| > 0.25 (FC, fold change) were set as the thresholds. We characterized the identities of these cell clusters based on the expression of canonical markers: *EPCAM*, *KRT19*, and *KRT7* for tumor cell; *COL1A1* and *ACTA2* for CAF; *CD3D* and *CD3E* for T cell; *CD68*, *CD14*, and *AIF1* for macrophage [12,16,17].

Inferred copy number variation analysis was carried out using inferCNV (inferCNV of the Trinity CTAT Project: https://github.com/broadinstitute/inferCNV, accessed on 10 April 2022). Normal epithelial cells from the GSA (CRA001160 [12]) were used as reference cells. For the inferCNV analysis, the following parameters were used: cut off = 0.1, and HMM_type = ‘i6’. As a result, heatmaps were generated to illustrate the relative expression intensities across each chromosome.

### 4.3. Re-Clustering of Tumor Cell to Explore Heterogeneity

Re-clustering of tumor cells was performed to refine the sub-populations with distinct metastatic risk. Genes expressed in fewer than 3 tumor cells were removed. Further analysis included normalizing and scaling the data, identifying HVGs, batch correction, and re-clustering. The functions used were the same as for cell type annotation. We re-clustered tumor cells at varying resolutions from 0.1 to 1 by the steps of 0.1. Here, we evaluated the clustering result at different resolutions through silwidth [20], which was a measure to estimate the similarity of cells within a cluster and that of cells between different clusters. A larger value of silwidth indicated that the cells of same cluster had a higher similarity. The average silwidth at each resolution was calculated using the cluster.stats function of fpc (V2.2.9) R package.

### 4.4. Metastatic Risk Estimation of Tumor Cell Sub-Clusters

To estimate the metastatic risk of tumor cell sub-clusters, we proposed a metastatic risk score, named scMetR, for each cell. Each cell was scored using the following formula [55]:

(1) $$X_{m}=S_{EMT}+S_{CyTo}$$

(2) $$M_{X_{m}}=\mathrm{mean}(S_{EMT})+\mathrm{mean}\left(S_{CyTo}\right)$$

(3) $$SD_{X_{m}}=\sqrt{SD_{S_{EMT}}^{2}+SD_{S_{CyTo}}^{2}+2\times r\times SD_{S_{EMT}}\times SD_{S_{CyTo}}}$$

(4) $$Score_{m}=\frac{X_{m}-M_{X_{m}}}{SD_{X_{m}}}$$

where $S_{EMT}$ is the score calculated by ssGSEA [21] to estimate the expression of genes from an EMT-associated gene resource (dbEMT 2.0) [22]; $S_{CyTo}$ is the cell differentiation score computed by CyToTRACE [23,24]; and *r* is the Pearson correlation coefficient between $S_{EMT}$ and $S_{CyTo}$. Both $S_{EMT}$ and $S_{CyTo}$ were scaled to [0, 1]. The Wilcoxon test was performed to calculate the significant difference between each tumor sub-cluster and the remaining tumor sub-clusters. *p* < 0.01 was set as the threshold. Based on the scMetR score, three tumor cell sub-populations with differing metastatic risks were defined, including MFTC, TransMTC, and ConvTC.

### 4.5. Functional and Survival Analysis Revealing Metastatic Risk of MFTC

The FindMarkers function was used to identify DEGs between MFTC and ConvTC, which were defined as MSGs. Adjusted *p* < 0.05 and |logFC| > 0.25 were set as the thresholds. Functional enrichment analysis was performed on the biological process (BP) category of Gene Ontology (GO) by using clusterProfiler (V4.2.1) R package [56]. The terms with minimum count ≥ 1 and adjusted *p* < 0.05 were selected.

We fetched bulk RNA-seq data of PDAC patients from TCGA (http://portal.gdc.cancer.gov/, accessed on 8 April 2022) as well as the AU and CA cohorts of the ICGC (http://dcc.icgc.org/, accessed on 8 April 2022). The patients without survival data were filtered. Finally, the number of samples retained for survival analysis was 146 in TCGA, 90 in AU cohort of ICGC, and 215 in CA cohort of ICGC. We used ssGSEA to estimate the up-regulated MSGs expression levels of patients. Patients were classified into high- or low-risk groups based on ssGSEA score. The surv_cutpoint function of survminer (V0.4.9) R package was used to determine the cut-off value, and the *p* value was calculated using the log-rank test.

### 4.6. Analysis of Spatial Transcriptomics Data to Discover Spatial Characteristic

We accessed public ST data of PDAC from the GEO database (GSE111672 [31]), which contained two primary samples. The PDAC-A sample contained 428 spatial spots, and PDAC-B contained 224 spatial spots. Genes expressed in fewer than 5 spots were removed. We used NormalizeData function to normalize data, RunPCA function to perform PCA, FindNeighbors and FindClusters to cluster the ST spots. Each cluster was annotated based on histological features [31].

To reveal the distribution of MFTC, we combined the scRNA-seq data by conditional autoregressive-based deconvolution (CARD) [57]. CARD required scRNA-seq with the gene expression information of distinct cell types along with ST data with localization information. The CARD performed deconvolution through a non-negative factorization framework and output the estimated cell-type composition across spatial locations with two inputs, which included the single-cell transcriptional profiles of all cell types in PDAC and the ST data of two PDAC samples. The CARD_deconvolution function of CARD (V1.0) R package was utilized to calculate the proportion of cell types at each spatial location [57].

### 4.7. Uncovering Cell–cell Communication Patterns Contributing to Metastasis

To reveal the communication patterns between major cell types, we employed CellChat (V1.5.0) [38] to perform cell–cell communication analysis. A CellChat object was created with normalized data to infer cell communication between different cell types. CellChat computed the probability of biologically significant communication patterns by assessing and integrating the gene expression levels of the CellChat-curated human ligand receptor database. We followed the official workflow and applied the preprocessing steps, including the functions identifyOverExpressedGenes, identifyOverExpressedInteractions, and projectData. Then, we calculated the potential ligand-receptor interactions between cell types based on the functions computeCommunProb, computeCommunProbPathway, and aggregateNet. All functions were run with default parameters.

### 4.8. Prediction of Candidate Drugs for Reversing the Expression of MSGs

Datasets of transcriptional responses to treatment with drugs were downloaded from the LINCS (http://www.Linc-sproject.org/, accessed on 2 June 2022). For each drug, transcriptional responses were represented by the FC between the treated and untreated conditions. Different treatment conditions were reported for the drugs, such as different cell lines of pancreatic cancer, drug concentrations, and times of treatment. The FC between the treated and untreated conditions was calculated for each gene. Then, the ranking lists of the FC from different conditions treated with the same drug were merged using Prototype Ranked List (PRL), which was achieved based on a hierarchical majority-voting scheme. The up- or down-regulated genes across the ranking lists were at the top or bottom of merging rank list [58]. Next, we used GSEA [40] to test whether MSGs were present among the genes which were up- or down-regulated by a drug to assess its potential as an inhibitor of metastasis [20]. The up-regulated genes of potential inhibitors for metastasis would contain down-regulated MSGs. The PRL was implemented by RankMerging function of GeneExpressionSignature (V1.38.0) package [59]. The fgsea function of fgsea (V1.18.0) R package was utilized for GSEA. The candidate drugs were selected by setting a threshold of *p* < 0.05.

## Data Availability

The data presented in this study are openly available in GEO at https://www.ncbi.nlm.nih.gov/gds, reference numbers are GSE154778, GSE156405 and GSE111672; in GSA at https://ngdc.cncb.ac.cn/gsa/, reference number is CRA001160; in dbEMT 2.0 at http://dbemt.bioinfo-minzhao.org/; in TCGA at http://portal.gdc.cancer.gov/; in ICGC at http://dcc.icgc.org/; in LINCS at http://www.Linc-sproject.org/.

## Supplementary Materials

The following supporting information can be downloaded at: https://www.mdpi.com/article/10.3390/ijms232315020/s1.
