# Supplementary material for: Estimating Metastatic Risk of Pancreatic Ductal Adenocarcinoma at Single-Cell Resolution
Source: Int J Mol Sci. 2022 Nov 30;23(23):15020. doi: 10.3390/ijms232315020 (PMC9736800; doi:10.3390/ijms232315020)
Supplement: Supplementary file 1 [file ijms-23-15020-s001.zip › supplementary File.docx]

**Estimating Metastatic Risk of Pancreatic Ductal Adenocarcinoma at Single-Cell Resolution**

Sina Chen ^†^, Shunheng Zhou ^†^, Yu-e Huang, Mengqin Yuan, Wanyue Lei, Jiahao Chen, Kongxuan Lin, Wei Jiang ^*^

Department of Biomedical Engineering, Nanjing University of Aeronautics and Astronautics, Nanjing 211106, China

* Correspondence: weijiang@nuaa.edu.cn

† These authors contributed equally to this work


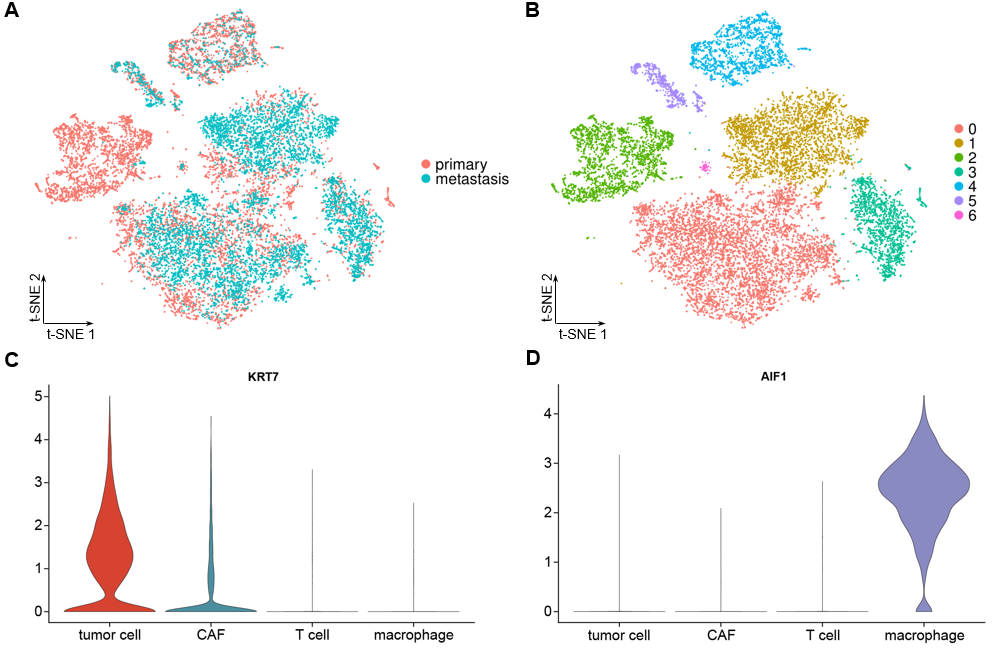


**Figure S1.** Cell clusters in the pancreatic ductal adenocarcinoma (PDAC) dataset. A-B. t-distributed stochastic neighbor embedding (t-SNE) plot of included cells for different conditions or cell clusters. Each color indicates one condition (A) or one cluster (B). C-D. Violin plots of expression of well-known markers. The y-axis shows the expression level.


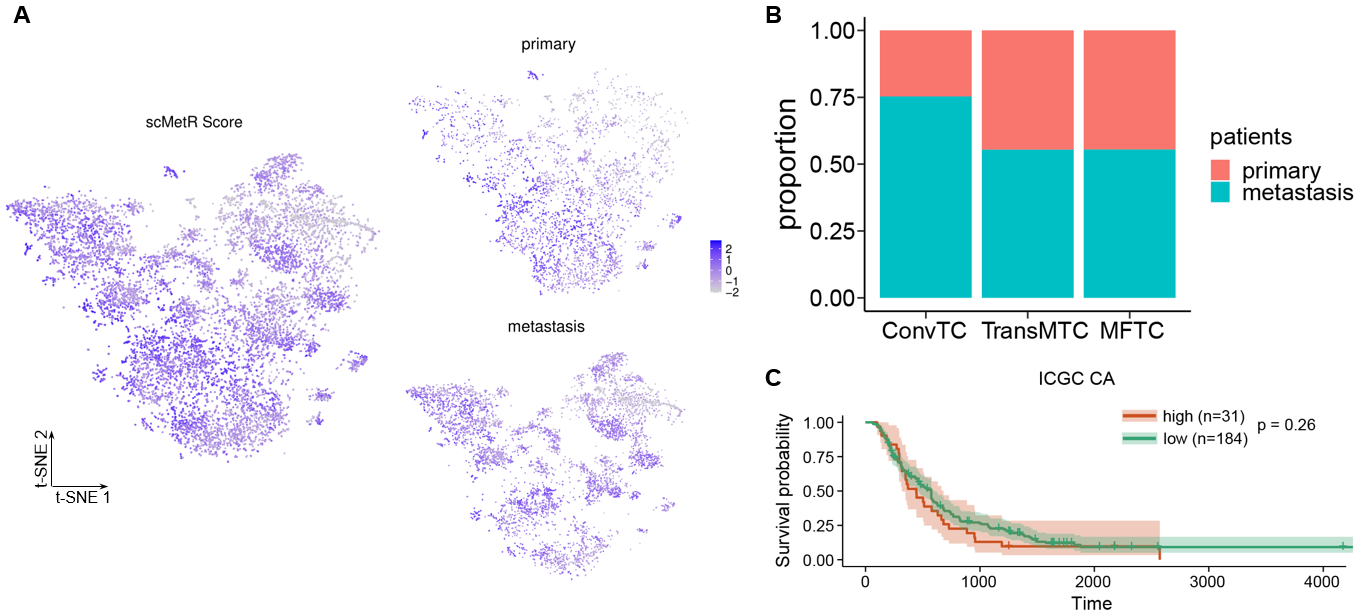


**Figure S2.** Analysis of the metastatic risk in tumor cells. A. The scMetR score of tumor cells, visualized by t-distributed stochastic neighbor embedding (t-SNE). The colors from grey to blue indicate the metastatic risk score from low to high, respectively. B. Bar plot of proportion in tumor sub-populations for primary and metastatic samples. C. Kaplan-Meier survival curve for patients in the CA cohort of International Cancer Genome Consortium (ICGC) dataset.


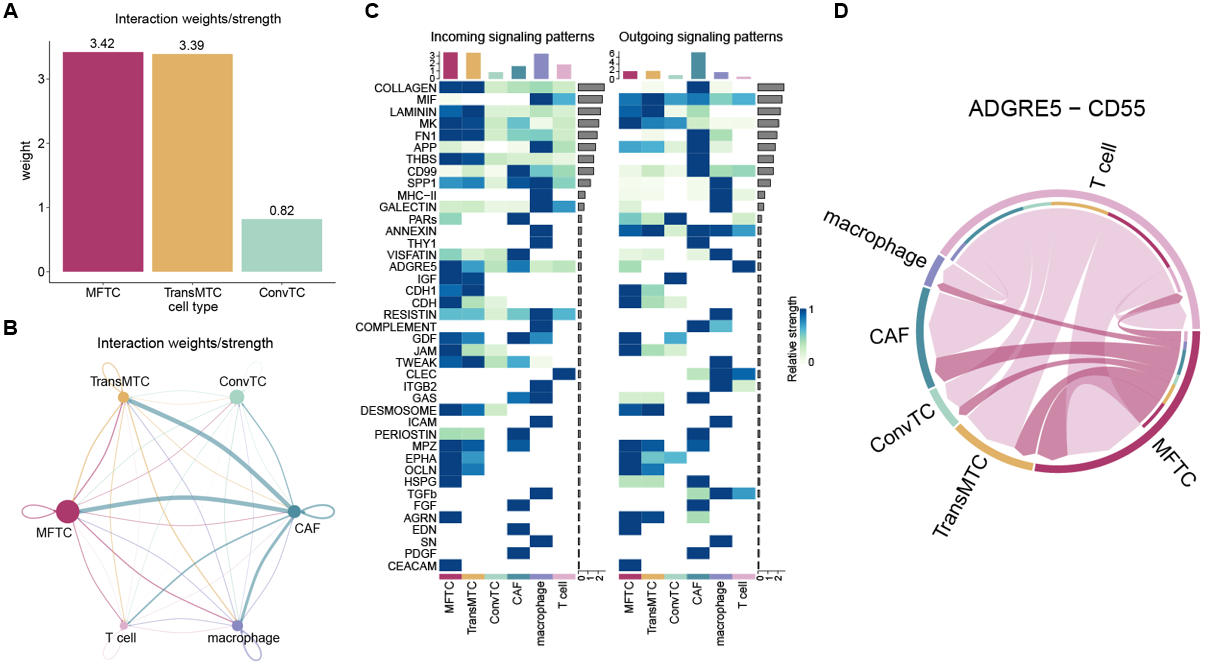


**Figure S3.** The cell-cell communication among all cell types. A. The total strength of interactions from tumor sub-populations. B. The strength of interactions among distinct cell types. The thickness of the lines connecting cells indicates the strength of the interactions. The size of the circles indicates the interaction strength of the cells. C. Heatmap of signaling changes in distinct cell types. The top bar plot represents the sum of incoming (left) or outgoing (right) signaling strength across all signaling pathways. The right bar plot represents the sum of incoming (left) or outgoing (right) signaling strength across all cell types. D. Chord plot of interactions in the ADGRE5 signaling pathway via ADGRE5 and CD55. ADGRE5 and CD55 are a ligand-receptor pair of the ADGRE5 signaling pathway.
