# Supplementary material for: Estimating Metastatic Risk of Pancreatic Ductal Adenocarcinoma at Single-Cell Resolution
Source: Int J Mol Sci. 2022 Nov 30;23(23):15020. doi: 10.3390/ijms232315020 (PMC9736800; doi:10.3390/ijms232315020)
Supplement: Supplementary file 1 [file ijms-23-15020-s001.zip › supplementary Note.docx]

**Estimating Metastatic Risk of Pancreatic Ductal Adenocarcinoma at Single-Cell Resolution**

Sina Chen ^†^, Shunheng Zhou ^†^, Yu-e Huang, Mengqin Yuan, Wanyue Lei, Jiahao Chen, Kongxuan Lin, Wei Jiang ^*^

Department of Biomedical Engineering, Nanjing University of Aeronautics and Astronautics, Nanjing 211106, China

* Correspondence: weijiang@nuaa.edu.cn

† These authors contributed equally to this work

Supplementary Note

In order to verify the efficiency of scMetR, we downloaded another pancreatic ductal adenocarcinoma (PDAC) single-cell RNA sequencing (scRNA-seq) dataset from Gene Expression Omnibus (GEO) database (GSE156405 [1]). The patients who had not been treated with any drugs were used for further analysis, which contained 4 primary patients and 1 metastatic patient. The number of cells obtained from each patient ranged from 1465 to 7479 for primary samples. The dataset included 14,124 cells of primary samples and 10,927 cells of metastatic sample. Genes expressed in fewer than 3 cells were removed. Cells with more than 40% mitochondrial gene counts, more than 6000 detected genes, and more than 75,000 unique molecular identifier (UMI) were filtered. Following quality control, we clustered 10,788 cells from primary samples and 9888 cells from metastatic sample based on gene expression profiles, and assigned cell types based on marker genes expression. Main cell types were assigned using the following marker genes: *EPCAM*, *KRT19*, and *KRT7* for tumor cell; *COL1A1* and *ACTA2* for cancer associated fibroblast (CAF); *CD3D* and *CD3E* for T cell; *CD68*, *CD14*, and *AIF1* for macrophage; *VWF*, *CLDN5*, and *EMCN* for endothelial cell; *MS4A1*, *CD79A*, and *CD79B* for B cell; and *CD1C* and *FCER1A* for dendritic cell [2-4]. Finally, the cells were annotated as B cell, CAF, dendritic cell, endothelial cell, macrophage, T cell, and tumor cell (Figure S4A).

To identify tumor sub-populations with different metastatic risk, we re-clustered the tumor cells, and observed that tumor cells were separated into six sub-clusters at the resolution of 0.1 (Figure S4B). The scMetR scores of the sub-clusters 0, 1, 2, 4, and 5 were significantly different from others (Wilcoxon test: P < 0.01). Thus, we defined sub-clusters 0, 2, and 5 as metastasis-featuring tumor cells (MFTC); sub-cluster 3 as transitional metastatic tumor cells (TransMTC); sub-clusters 1 and 4 as conventional tumor cells (ConvTC). We compared the scMetR scores among tumor sub-populations, and found that the scMetR score was also significantly enhanced in MFTC compared to TransMTC, and in TransMTC compared to ConvTC (Figure S4C).

To verify the metastatic risk of MFTC, we performed further analysis. We applied functional enrichment analysis on the up-regulated metastatic signature genes (MSGs), and found that the up-regulated MSGs were also enriched in the functions related to metastasis, such as neutrophil degranulation [5] and neutrophil mediated immunity [6]. The results of survival analysis displayed that the patients with high expression of the up-regulated MSGs had worse prognosis as well (Figure S4D-E). In addition, the up-regulated MSGs contained some genes which were verified to promote PDAC metastasis, such as *HMGA1* [7]. Spatial transcriptomics (ST) analysis revealed that *HMGA1* was highly expressed in duct epithelium region and cancer region (Figure S4F). After mapping cell types in PDAC slides, we observed that MFTC was preferentially located in duct epithelium region and cancer region (Figure S4G). Further, cell-cell interactions were inferred by CellChat. The total number and the strength of interactions were the highest in MFTC and second highest in TransMTC (Figure S4H). These results were consistent with the discoveries of Lin *et al* data [3], which suggested that scMetR was reproducible in the independent dataset.


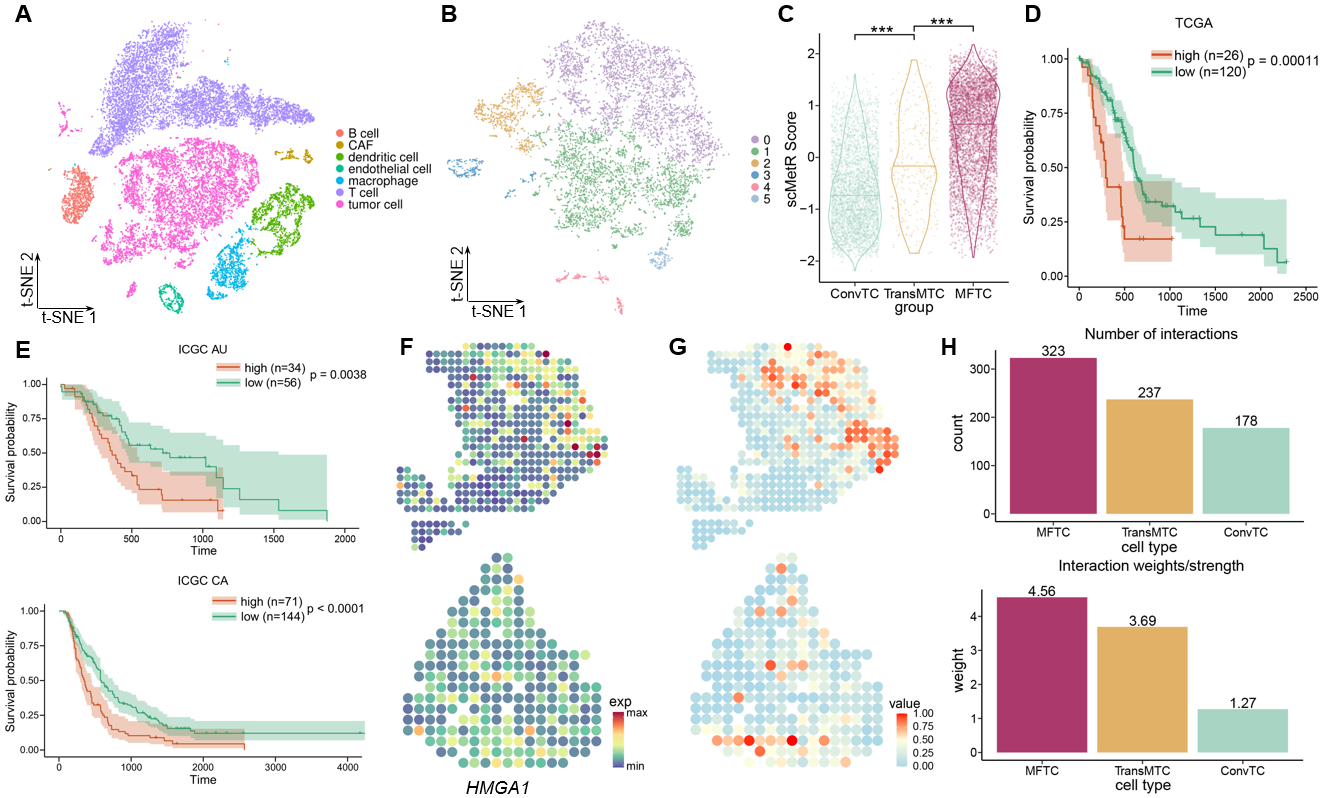


**Supplementary Figure S4**. The analysis of single-cell RNA sequencing (scRNA-seq) validation dataset. A. t-distributed stochastic neighbor embedding (t-SNE) plot of 7 major cell types identified in primary and metastatic pancreatic ductal adenocarcinoma (PDAC). B. t-SNE plot of tumor sub-clusters at the resolution of 0.1. C. Violin plots of the scMetR scores for tumor sub-populations. D-E. Kaplan-Meier survival curves for patients in The Cancer Genome Atlas (TCGA) (D) as well as the AU and CA cohorts of International Cancer Genome Consortium (ICGC) (E). F. Spatially resolved heatmaps of expression patterns of *HMGA1*. G. Spatially resolved heatmaps of the proportion of metastasis-featuring tumor cells (MFTC). The proportion is encoded by the colors. H. Total number (top) and strength (bottom) of the cell-cell interactions of the tumor sub-populations.

References

1. Lee, J.J.; Bernard, V.; Semaan, A.; Monberg, M.E.; Huang, J.; Stephens, B.M.; Lin, D.; Rajapakshe, K.I.; Weston, B.R.; Bhutani, M.S.; et al. Elucidation of Tumor-Stromal Heterogeneity and the Ligand-Receptor Interactome by Single-Cell Transcriptomics in Real-world Pancreatic Cancer Biopsies. *Clin. Cancer Res.* **2021**, *27*, 5912–5921. https://doi.org/10.1158/1078-0432.CCR-20-3925.

2. Peng, J.; Sun, B.F.; Chen, C.Y.; Zhou, J.Y.; Chen, Y.S.; Chen, H.; Liu, L.; Huang, D.; Jiang, J.; Cui, G.S.; et al. Single-cell RNA-seq highlights intra-tumoral heterogeneity and malignant progression in pancreatic ductal adenocarcinoma. *Cell Res.* **2019**, *29*, 725–738. https://doi.org/10.1038/s41422-019-0195-y.

3. Lin, W.; Noel, P.; Borazanci, E.H.; Lee, J.; Amini, A.; Han, I.W.; Heo, J.S.; Jameson, G.S.; Fraser, C.; Steinbach, M.; et al. Single-cell transcriptome analysis of tumor and stromal compartments of pancreatic ductal adenocarcinoma primary tumors and metastatic lesions. *Genome Med.* **2020**, *12*, 80. https://doi.org/10.1186/s13073-020-00776-9.

4. Pan, H.; Diao, H.; Zhong, W.; Wang, T.; Wen, P.; Wu, C. A Cancer Cell Cluster Marked by LincRNA MEG3 Leads Pancreatic Ductal Adenocarcinoma Metastasis. *Front. Oncol.* **2021**, *11*, 656564. https://doi.org/10.3389/fonc.2021.656564.

5. Charles Jacob, H.K.; Charles Richard, J.L.; Signorelli, R.; Kashuv, T.; Lavania, S.; Vaish, U.; Boopathy, R.; Middleton, A.; Boone, M.M.; Sundaram, R.; et al. Modulation of Early Neutrophil Granulation: The Circulating Tumor Cell-Extravesicular Connection in Pancreatic Ductal Adenocarcinoma. *Cancers* **2021**, *13*, 2727. https://doi.org/10.3390/cancers13112727.

6. Wang, X.; Hu, L.P.; Qin, W.T.; Yang, Q.; Chen, D.Y.; Li, Q.; Zhou, K.X.; Huang, P.Q.; Xu, C.J.; Li, J.; et al. Identification of a subset of immunosuppressive P2RX1-negative neutrophils in pancreatic cancer liver metastasis. *Nat. Commun.* **2021**, *12*, 174. https://doi.org/10.1038/s41467-020-20447-y.

7. Kajioka, H.; Kagawa, S.; Ito, A.; Yoshimoto, M.; Sakamoto, S.; Kikuchi, S.; Kuroda, S.; Yoshida, R.; Umeda, Y.; Noma, K.; et al. Targeting neutrophil extracellular traps with thrombomodulin prevents pancreatic cancer metastasis. *Cancer Lett.* **2021**, *497*, 1–13. https://doi.org/10.1016/j.canlet.2020.10.015.
